# Tipping the scales: the predictive utility of the PCE-ACE ratio for criminogenic and wellbeing outcomes in a general adult population

**DOI:** 10.1186/s40352-026-00397-1

**Published:** 2026-01-28

**Authors:** Colm Walsh

**Affiliations:** SSESW, Queen’s University Belfast, Belfast, United Kingdom

**Keywords:** ACEs, Adversity, Arrest, Crime, Mental health, Offending, PCEs, Positive Childhood Experiences, Wellbeing

## Abstract

**Background:**

Adverse Childhood Experiences (ACEs) and Positive Childhood Experiences (PCEs) are each independently associated with a range of adult outcomes, including mental health, substance use, and criminal justice involvement. However, few studies have examined how the balance between these experiences influences outcomes. This study explores the predictive utility of a PCE:ACE ratio. Unlike previous measures of resiliency and risk protection scales that treat risk and protective factors as parallel dimensions, the ratio is population-level heuristic intended to capture the relative balance of positive versus adverse experiences using a single relational metric. Using data from a representative sample of 1,203 adults in Northern Ireland, participants completed validated measures of 13 ACEs and 10 positive childhood experiences (PCEs) A weighted PCE:ACE ratio was calculated, and participants were categorised into high, moderate, or low ratio groups.

**Results:**

Findings showed that a higher ratio was significantly associated with reduced odds of arrest, incarceration, school exclusion, substance use, and mental health diagnosis, even after adjusting for age, gender, and deprivation. Those in the low-ratio group had the highest rates of adverse outcomes. While the ratio offers an intuitive and accessible framework for understanding developmental balance, limitations include the potential for oversimplification of distinct ACE-PCE profiles.

**Conclusions:**

These findings support the feasibility of a ratio-based approach that standardises balance rather than the independent accumulation of risks and strengths, and suggests that a stronger balance of protective experiences may buffer the impact of adversity. Further research is needed to explore threshold effects and interaction dynamics. However, the ratio provides a useful metric and sound basis for capturing population health and the extent to which public investment is tipped in favour of positive or less positive outcomes.

## Introduction

### ACEs

Adverse Childhood Experiences (ACEs) refer to a collection of potentially traumatic events experienced during childhood but remembered into adulthood (Ports et al., 2019). While research on child maltreatment spans more than fifty years (Madigan et al., 2023), the seminal ACE study by Vincent Felitti and colleagues (Felitti et al., 1998) highlighted the extent of childhood abuse, neglect, and family dysfunction among adults in the United States. This study catalysed a global surge in ACE research (Hughes et al., 2017), consistently illustrating the prevalence of adversity in the general population. Although estimates vary across contexts and research designs (Pace et al., 2022), the pooled global prevalence of exposure to at least one ACE is approximately 60%, and 16.1% for exposure to four or more ACEs (Madigan et al., 2023). In the EU, the prevalence of four or more ACEs is estimated at 5.6% (Hughes et al., 2019). The growth in ACE studies has been instructive, providing a shared language (Devaney et al., 2021) that has deepened our understanding of the range of harms that children may experience (Kessler et al., 2010)- harms that have been implicated in a wide range of deleterious outcomes, including those that are criminogenic (Baglivio et al., 2014, educational (Stewart-Tufescu et al., 2022), behavioural and mental health outcomes (Kessler et al., 2010).

### ACE outcomes

Across the ACE literature, it has been more common than not to compare those with no ACEs with individuals reporting multiple ACEs (e.g., four or more) (Madigan et al., 2023), with findings favouringa dose–response effect. Those in the higher ACE groups are at significantly greater risk of being excluded from school (Stewart-Tufescu et al., 2022), developing diagnosable mental health conditions (McLaughlin, Weissman & Brian, 2019; Abate et al., 2025) and coming into contact with the criminal justice system (Astridge et al., 2023). Indeed, it is estimated that between 75% and 93% of children entering the juvenile justice system report that they have experienced at least one traumatic event (Branson et al., 2017), and up to 80% of violent offenders have been victims themselves (Maschi and Bradley, 2008), a figure that holds across continents. What the ACE research focuses on, is the clustering effect of adversity (Madigan et al., 2023). That is, those who experience one are more likely to experience multiple (Dong et al., 2004) and those who experience the highest number of ACEs are at greatest risk of such outcomes (Hughes et al., 2017). Some estimate that for each additional ACE, there is a 12.9% increase in the risk of multi-morbidity (Senaratne et al., 2024), and that for each point on the ACE measure, there is a corresponding increase in the odds of justice system contact (Graf et al., 2021). In their systematic review and meta-analysis of exposure to multiple ACEs and health outcomes, Hughes et al. (2017) found that despite being at greater risk of a range of outcomes, ranging from obesity to cancer to sexually transmitted infections, the strongest effects were observed for problem alcohol use (OR = 5.84 (3.99–8.56)), violence victimisation (OR = 7.51 (5.60–10.08)), violence perpetration (OR = 8.10 (5.87–11.18)), and problem drug use (OR = 10.22 (7.62–13.71)). In recently published prevalence studies it was observed that compared with those in the lowest ACE group (0 ACEs), those who reported four or more ACEs were almost ten times more likely to report a mental health diagnosis (OR = 9.56 (6.21–14.72)), almost nine times more likely to have been excluded from school as a child (OR = 8.96 (4.64–17.31)), and more than 8 times more likely to have ever been arrested (OR = 8.3 (4.86–14.10)) (Walsh et al., 2025a, 2025b). However, these figures, like previous ACE studies belie an important observation, not everyone who experiences ACEs, even in multiples, will experience these outcomes.

Recent research has increasingly examined mechanisms underlying ACE-outcome relationships. Reviews show that some adversities, particularly violence-related ones, are more harmful (Hughes et al., 2021); some groups, such as females, are at greater risk of specific ACEs (Haar-Pederson et al., 2020); and that outcomes differ, even among individuals exposed to the same events, with deprivation emerging as a key moderator (Merrick et al., 2020); Madigan et al., 2023). Despite these insights, gaps remain. Specifically, why do some individuals experience negative outcomes, yet others, exposed to exactly the same types of events, do not?

### Positive Childhood Experiences (PCEs)

To complement the ACE research, there has been a growth of interest in what happens to whom and why following adversity (Merrick & Narayan, 2020; Kallapiran et al., 2025), and what the specific assets or resources that promote recovery and that facilitate healthy development are (Han et al., 2024). One line of inquiry has been the role of advantageous experiences or the counter-ace paradigm (Crandall et al., 2019; Novak & Fagan, 2022; Cunha et al., 2024). These Positive Childhood Experiences (PCEs) are characterised by safety and security, positive self-perceptions, and social support (Narayan et al., 2018). If ACEs are the types of experiences that we would not expect from a normal environment, conducive to healthy development, PCEs are favourable life events and experiences that impact positively on developmental and life course outcomes, including mental health and wellbeing (Lamb & Lerner, 2015). They are concerned with the familial and extra-familial factors (Walsh et al., 2025b) that include but extend beyond support. They are aligned to and even complement earlier theories of resilience (Masten, 2001) which provides what Crandall et al., (2019: 2) refers to as the *‘…logical theoretical frameworks for this study of counter-ACEs’.* With its roots in the 1970 s, early resiliency researchers began to explore why, given the same types of exposure that were predicted to lead to negative outcomes (Wolke et al., 2025), many people did better than expected with regard to educational, mental health and physical health outcomes (Masten, 2007). This seminal and somewhat unexpected observation had potential for significant policy impact (Cutuli et al., 2021), and while definitions varied (as they still do) (Wolke et al., 2025), there was emerging consensus in the decades that followed that resilience extended beyond individual traits to capture the capacity of systems to adapt to the types of difficulties that threaten positive functioning and development (Masten, 2014). This aligned at the same time with more relational ideas offered by criminological and relational theories of positive youth development (PYD) such as social support (Cullen, 1994; Ungar, 2013). Despite instructive research on resiliency in the 1990 s, research found limitations that require novel advances in conceptualisation and measurement. For instance, across the literature, definitions of resilience varied. When it was defined, the factors that produced or inhibited resilience were conceptualised as opposite factors on a pole and variables that spanned innate traits, dispositions, genetic dispositions, as well as relational, systemic and cultural factors (Cutuli et al., 2021). This diversity has made measurement and standardisation difficult (Wolke et al., 2025). Ungar (2013) has also noted that the literature has tended to treat these clustering of resilience producing and resilience inhibiting factors as parallel variables, when in fact observations implied that both positive (e.g., support) and negative (threat) experiences could co-occur. Further, much of the resilience research has also tended to treat positive and negative experiences as additive (Cutuli et al., 2021). The assumption is that more of the good equals less of the bad without consideration of the interaction between the two (Rutter, 2006). However, the emergence of research into what is broadly referred to as positive childhood experiences (PCEs), while building on the literature around resilience and social support, has also been a departure. While protective factors include a broad array of internal resources and external supports (Wolke et al., 2025), not all protective factors are PCEs. The PCE research has also provided opportunities to standardise definitions, conceptualisation, and measurement of exposures, positive and negative, at a population level.

Research over the previous decade has also shown that just like ACEs, those who experience multiple PCEs are more likely to experience more positive mental health (Bethell et al., 2019), reduced levels of offending (Baglivio & Wolff, 2021; Kowalski et al., 2022), and improved educational outcomes, even when they are exposed to adversity. Explaining these observations has proved more difficult, with the research on the specific contribution of PCEs and interaction with ACEs (or not) to improving outcomes being largely inconsistent (Bunting et al., 2023; Crandall et al., 2019). While some studies have illustrated the protective nature of PCEs (i.e., that they indirectly contribute to positive outcomes via a buffering effect on ACEs (Morris et al., 2021), others have found no evidence for the protective mechanism (Bunting et al., 2023; Crandall et al., 2019; Han et al., 2023). Instead, several authors have proposed that there is a promotive effect, which shows the importance of positive childhood experience independent of, and even in the absence of, ACEs (Zimmerman, 2013; Han et al., 2023). In other words, PCEs facilitate healthy development and positive outcomes without the need for ACEs being present. Despite the focus on the protective or promotive nature of PCEs, their interaction with ACEs and contribution to positive/negative outcomes, most studies have tended to focus on one (e.g., PCEs) and control for the other (e.g., ACEs) (Narayann et al., 2018; Charite et al., 2023).

Another lens through which to understand the impact of both ACEs and PCEs on outcomes, however, has been through understanding the relationship between the two. One common way of conceptualising this has been via the Challenge Model (Fleming & Ledogar, 2008). According to the Challenge Model, the relationship between the ACEs and PCEs is not linear, but curvilinear. Through this lens, moderate experiences of stress enhance the capacity to ‘bounce back’ and to recover more quickly from adversity (Masten, 2001). Another way of conceptualising the link between the two is that rather than merely having exposure to either/or, there may be a *‘tipping point’* where the cumulative effect of one is significant enough to move the balance of probabilities towards either a more negative or more positive outcome. Villadsen, Libuy & Fitzsimons (2025) for instance, found that 19.5% of their sample (age 17) had experienced 4 + ACEs, but on their measure of 7 PCE’s, the average number was 3.8. They hypothesised (but did not test the idea) that rather than a relatively simple graded analysis, with a cut-off for either ACEs or PCEs, a ratio could be a useful departure. In one of the few studies examining ACEs and PCEs and their effect on five health outcomes, Crandall et al. (2020) found that where PCEs outweighed ACEs, health outcomes were more positive. Additionally, *‘…this study included a cumulative measure of advantageous experiences (counter-ACEs) that can be used to compare against cumulative adverse events…when ACEs and counter-ACEs were considered together, ACEs were not associated with any of the negative health outcomes…’* (Crandall et al., 2020: 5). The authors speculated that PCEs could have a direct effect only when they outweigh ACEs, proposing that despite the growing evidence, more research is required to test new ways of understanding the interaction between the two.

### Aims

The aim of the current study is to: (1) explore the feasibility of developing a PCE:ACE ratio for understanding the links between positive childhood experience, adverse childhood experiences and outcomes; (2) compare high, moderate and low PCE:ACE ratio groups with criminogenic and wellbeing outcomes; (3) model the predictive utility of the ratio of these outcomes using binary logistic regression.

## Methods

### Study design and setting

Data analysed in this study are based on the NI ACEs Survey which used a stratified random probability sampling design to assess childhood adversity among over 1,200 adults aged 18 + years in Northern Ireland (see Walsh et al., 2025a, 2025b).

### Measures

#### Positive Childhood Experiences (PCEs)

PCEs were assessed using a 10-item scale developed from the Benevolent Childhood Experiences (PCEs) framework (Narayan et al., 2018), assessing supportive relationships, community connectedness, school belonging, and perceptions of safety. Each item was scored as 0 (No) or 1 (Yes)**,** with a cumulative range of 0 to 10**.**

#### Adverse Childhood Experiences (ACEs)

ACEs were measured using a 13-item scale adapted for conflict-affected contexts, including abuse, neglect, household dysfunction, community violence, and loss. Childhood adversity was measured using the WHO ACE-IQ (2011), adapted for NI. The ACE-IQ measure was developed by consensus via the World Health Organisation 2011) to provide a standardised way of capturing a wider set of adversities (abuse; parents; family dysfunction; extra-familial violence) via 13 categories, some of which are not routinely captured using the original ACE tool (Felliti et al., 1998). Responses can be dichotomised (1 = present; 0 = absent), or captured as higher frequency experiences (e.g., ‘many times’). The 13-item tool captures abuse, family dysfunction, and Extra-familial Violence (EFV). Items used in the current study were based on higher frequency exposure. The measure has good reliability (α =.85) and predictive validity (R^2^ =.12) (Christoforou & Ferreira, 2020).

#### Warwick Edinburgh Mental Well-being Scale (SWEMWBS)

The short 7-item version of the Warwick Edinburgh Mental Well-being Scale (Stewart-Brown et al., 2009) was used to measure mental well-being in adults.

#### The Patient Health Questionnare-4 (PHQ-4)

Kroenke et al., 2009 was used as a concurrent measure of anxiety and depression symptoms.

#### DUDIT

Problem Substance use was measured using a combination of substances listed in the DUDIT screening tool (Berman et al., 2005) and the Northern Ireland Wellbeing survey (Bunting et al., 2020).

#### Client Service Receipt Inventory (CSRI)

Were used to capture criminal justice system contact, including arrest and incarceration. To capture academic engagement, a single item with binary response asked if respondents whether ‘during childhood, you were ever formally excluded from school (e.g., suspended or expelled)?

### Data preparation

To account for the unequal maximum scores of the PCE (10) and ACE (13) scales, a weighted adjustment was applied to the PCEs:

### Weighted PCE Score = Total PCEs × 1.3

A PCE to ACE ratio was then computed for each participant. A small constant (0.0001) was included in the denominator to avoid division by zero when no ACEs were reported. This provided a raw PCE to ACE ratio:

### PCE: ACE Ratio = Weighted PCE Score/(Total ACE Score + 0.0001)

Using this ratio, participants were also categorised into three groups. Participants were stratified into groups based on their weighted PCE:ACE ratio, calculated to reflect the balance of positive and adverse childhood experiences. Cut-off points were conceptually grounded in resilience theory, where a ratio of 1.0 reflects balance, < 1.0 indicates more adversity than protection, and ≥ 4.0 represents substantial protective buffering. These thresholds also aligned with the observed distribution of ratios in the sample.

- High PCE:ACE ratio (≥ 4.0)
- Moderate PCE:ACE ratio (1.0 to 3.99)
- Low PCE:ACE ratio (< 1.0)

### Data analysis

Descriptive statistics were used to explore the distribution of PCE and ACE scores, ratios, and high, moderate, and low groups. Chi-square tests were used to explore differences between groups (high PCEs to ACE ratio compared with those who had lower PCE to ACE ratio) with categorical outcomes (e.g., mental health and wellbeing, educational exclusion, substance use and arrest).One-way Anova tests were used to explore mean differences between groups with high PCEs: ACE ratios compared with those who had lower PCE: ACE ratios with categorical outcomes (e.g., wellbeing).Binary logistic regression models were conducted to assess the predictive power of the PCE:ACE ratio on key criminogenic and wellbeing outcomes. Because of the risk of extreme values due to zero, ACEs within the sample, and the potential for a right-skewed distribution, the ratio was log-transformed as follows:

### Log-transformed Ratio = log10 (PCE: ACE Ratio + 1)

This transformation normalised the distribution and allowed for more stable coefficient estimation in regression models. All analyses were performed using SPSS (Version 29.0), with statistical significance set at *p* < 0.05.

## Findings

### Sample characteristics

A total of *N* = 1203 adults participated in the study. 44% of the sample were male and 56% were female. There were no statistically significant gender differences between this sample and the Northern Ireland population. On average, participants were 49.58 years old (SD = 17.62). The mean number of Positive Childhood Experiences (PCEs) was M = 8.6 (SD = 1.72) and the mean number of Adverse Childhood Experiences (ACEs) was M = 1.73 (SD = 2.32)**.** After weighting, the log-transformed PCE-ACE ratio mean was M = 2.5 (SD = 2.12) indicating a high degree of protective experiences relative to adversity in the sample.

Using the raw ratio score, participants were grouped into:

- High PCE:ACE ratio (≥ 4.0): *n* = 911 (75.7%)
- Moderate PCE: ACE ratio (1.0–3.99): *n* = 234 (19.5%)
- Low PCE: ACE ratio (< 1.0): *n* = 58 (4.8%)

While previous studies note the importance of deprivation on likelihood of ACE exposure, analysis revealed a statically significant difference between High and Low PCE:ACE ratio between the most and least deprived areas (*X*2 (8, *N* = 1203) = 26.73, *p* = <.001). Specifically, those in the low PCE:ACE ratio group were more likely to be from the most deprived areas (25.9%) compared with the least deprived (6.9%), implying that there the balance favours ACEs over PCEs in the most deprived area. However, the difference between those in the high PCE:ACE ratio group is less divergent (16.9% v 22.7%) implying that while those in the least deprived areas remain more likely to experience higher positive than adverse experiences, the difference is more marginal.

### Group differences in criminogenic and wellbeing outcomes

**Fig. 1:**
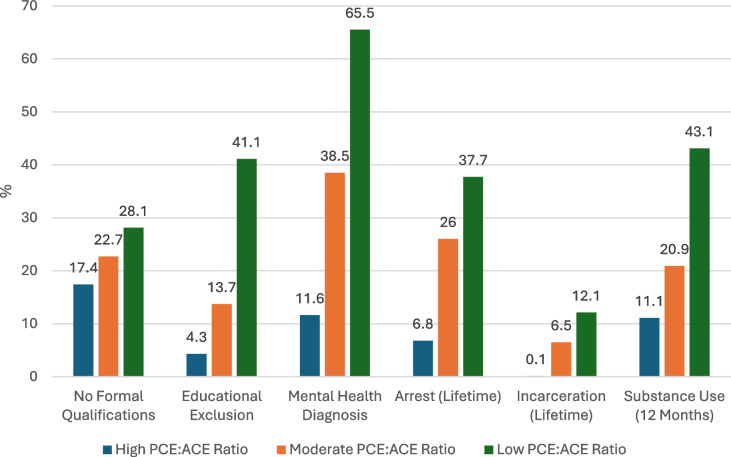
% Criminogenic/wellbeing outcomes by group

**Fig. 2:**
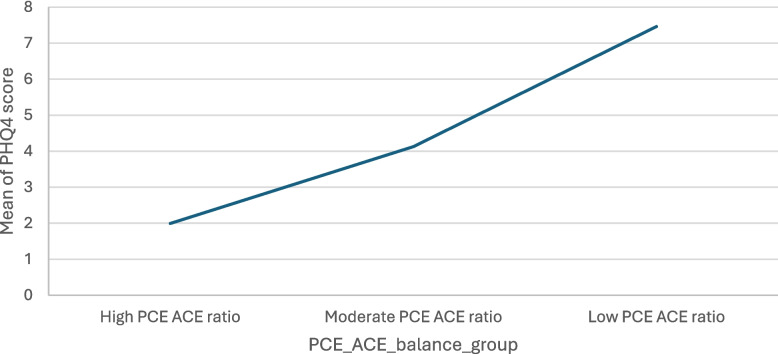
PCE:ACE ratio groups and mean PHQ scores

Chi-square tests were used to explore associations between the group category and the following binary outcomes. A visual inspection of Figure shows that across outcome areas, there are clear differences in reported exposure between low, moderate and high ratio groups. There were statistically significant differneces across all groups, including, no formal qualifications (${X}^{2}$ (2, *N* = 225), 6.21, *p* =.045), educational exclusion (${X}^{2}$ (2, *N* = 94), 112.41, *p* = <.001), any mental health diagnosis (${X}^{2}$ (2, *N* = 234), 168.05, *p* = <.001), arrest (${X}^{2}$ (2, *N* = 138), 72.9, *p* = <.001), and illicit drug use (${X}^{2}$ (2, *N* = 175), 54.52, *p* = <.001), with the distance between the lowest and the highest ratio groups most striking in the areas of mental health and criminal justice outcomes (Figs. 1 and 2).

### Group differences and demographic factors

While inconsistent findings have been observed, both the ACE and PCE literature illustrate differences in outcomes across demographic characteristics such as gender and deprivation. A series of chi-square tests explored these criminogenic and wellbeing outcomes, accounting for these characteristics. While the low numbers prevented analysis of educational attainment across the five deprivation quintiles, this outcome only remained significant for males (.004) while became non-significant for females (.68). 40% of males in low PCE:ACE group (*N* = 10) had no qualifications, while only 18.8% of females in the same group reported the same outcome (*N* = 6) (see Table 1). Males and females were equally likely to be excluded from school when the ratio of PCE:ACE favoured adversity, however, compared with 53% of those in low group in the most deprived areas, 0% of those in low PCE:ACE ratio group, and who were in least deprived were excluded. This suggests that for some outcomes, including school exclusion, other factors beyond the PCE:ACE ratio elevate risks, including school culture and discipline practices. There were no statistically significant gender differences regarding mental health issues, and neither were there any statistically significant difference on deprivation. In other words, a higher PCE:ACE ratio was equally protective for males, females and those from more or less affluent areas.

**Table 1:** Associations between PCE:ACE ratio groups and key outcomes by gender

	PCE/ACE Ratio Group	No qualifications % (n)	School exclusion % (n)	Drug use % (n)	Arrest % (n)	Incarceration % (n)
Male	Low	40 (10)	36 (9)	40 (10)	57.1 (12)	24 (6)
	Moderate	23.6 (26)	19.1 (21)	23.6 (26)	43.7 (45)	13.9 (15)
	High	16 (63)	6.6 (26)	13.2 (52)	13.9 (53)	1 (0.3)
	${X}^{2}$, *p*	11.04, *p* =.004	52.05, *p* = <.001	16.99, *p* = <.001	58.53, *p* = <.001	53.96, *p* = <.001
Female	Low	18 (6)	42.4 (14)	9.5 (49)	25 (8)	3 (1)
	Moderate	21.1(26)	8.9 (11)	17.9 (22)	10.1 (12)	0 (0)
	High	18 (93)	2.5 (13)	9.5 (49)	1.4 (7)	0 (0)
	${X}^{2}$, *P*	.655, *p* = 721	96.3, *p* = <.001	39.55, *p* =.001	55.64 (<.001)	6.06, *p* =.048

There were no statistically significant gender differences regarding past year drug use, with both males and females most likely to have reported experience in the lowest ratio group, however, compared with those in the least deprived areas, those in most deprived areas, and who were in the low PCE: ACE ratio group were most likely to report drug use (*p* =.004). Those in least deprived areas were the only one of the five quintiles where the association between ratio and substance use outcome was no longer significant (*p* =.472).

There were no statistically significant gender differences between males and females on arrest, however, the proportions varied, with 57.1% of males in the low PCE:ACE group reporting arrest, while this was the case for 25% of females. While all deprivation quintiles were significant at the *p* = <.001 level, those in the least deprived areas appeared to be most likely to experience arrest when in the low PCE:ACE group. Compared with those in the most deprived (28.6%), 66.7% of those in the least deprived group, who were in the low category, reported arrest.

PCE:ACE ratio was statistically significant for both males and females at the *p* = <.001 level, but only 3% of females in the low ratio group reported incarceration while 24% of males in low PCE:ACE ratio group reported the same outcome.

### Mental health

There was a statistically significant difference in the mean score of wellbeing and the PCE:ACE ratio groups *(F* (2, 1180) = 126.54, *p* = <.001). A Tukey post-hoc test revealed that those in the high PCE: ACE ratio group had significantly lower mental health needs (M = 1.99, SD = 2.58) compared with the moderate (M = 4.13, SD = 3.79) and low PCE:ACE ratio groups (M = 7.46, SD = 3.64).

### Wellbeing

**Fig. 3:**
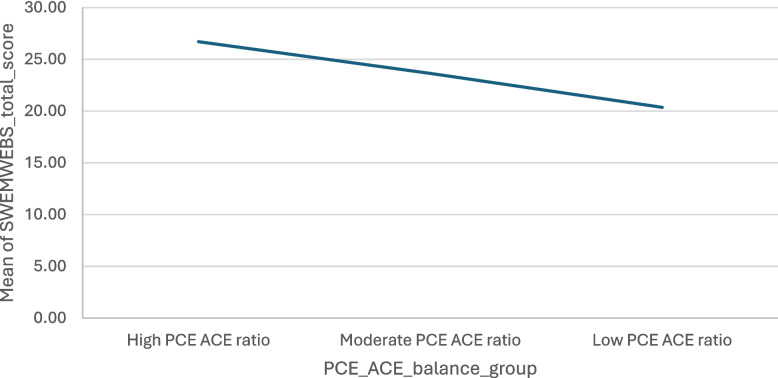
PCE:ACE ratio groups and mean wellbeing scores

There was a statistically significant difference in the mean score of wellbeing and the PCE:ACE ratio groups *(F* (2, 1200) = 1775.75, *p* = <.001) (see Fig. 3). A Tukey post-hoc test revealed that those in the high PCE: ACE ratio group had significantly higher wellbeing (M = 26.7, SD = 5) compared with the moderate (M = 23.64, SD = 5.93) and low PCE:ACE ratio groups (M = 20.36, SD = 6.59).

### Binary logistic regression

Binary logistic regression analyses were conducted to examine whether or not higher PCE:ACE ratios (log transformed) were associated with improved outcomes compared with those with lower PCE:ACE ratios. Given previous studies have illustrated significant differences on ACE and PCE outcomes, all analyses controlled for age, gender and deprivation. Each reduction in the OR reflects less positive outcomes.

The odds of being excluded from school were significantly lower for participants with higher PCE:ACE ratios (OR =.656, 95% CI [.563,.764], *p* =.001), suggesting that, despite experiencing adversity during childhood, a balance that favours positive childhood experiences is protective and that the higher that ratio of positive to adverse experiences is the likelihood of better outcomes that there is. Here, those with stronger positive childhood experiences were approximately 34% less likely to have been excluded from school. While the trend was in the expected direction for leaving school with no formal qualifications, the association between the PCE:ACE ratio was not statistically significant. Regarding health outcomes, a higher PCE:ACE ratio was associated with significantly reduced odds of any mental health diagnosis (OR =.683, 95% CI [.624,.748], *p* <.001), with the same protective nature observed for substance use outcomes (OR =.833, 95% CI [.764,.908], *P* <.001). Modelling contact with the criminal justice system, higher PCE:ACE ratios were predictive of reduced arrest (OR =.718, 95% CI [.644,.801], *p* <.001) and incarceration (OR =.015, 95% CI [.002,.098], *p* <.001). This shows that within this sample, those with higher PCEs were approximately 28% less likely to have been arrested and 98.5% less likely to have been incarcerated (Table 2).

**Table 2:** Results from PCE:ACE ratio binary logistic regression

Outcomes	Total		PCE:ACE ratio groups			Results from Binary Logistic Regression Comparing 4 + ACEs with 0 ACES						
			%^b^					Confidence Interval		Omnibus Tests of Model Coefficients		
	N	%	High	Moderate	Low	p	OR	Lower	Upper	***X***2	df	p
Education												
No formal qualifications	225	18.7	17.4	22.7	28.1	.082	.932	.861	1.009	232.19	4	<.001
School Exclusion	94	7.8	4.3	13.7	41.1	<.001	.656	.563	.764	91.86	4	<.001
Health												
Any mental health diagnosis	234	18.7	11.6	38.5	65.5	<.001	.683	.624	.748	125.31	4	<.001
Substance use	175	14.5	11.1	20.9	43.1	<.001	.833	.764	.908	81.97	4	<.001
Criminal behaviour												
Arrest	138	11.5	6.8	26	37.7	<.001	.718	.644	.801	154.55	4	<.001
Incarceration	23	1.9	0.1	6.5	12.1	<.001	.015	.002	.098	103.66	4	<.001

^a^Adjusted for age, gender and deprivation; b these percentages reflect the distribution of each outcome variable within in each of the three groups

## Discussion

The ACE research, and more recently, the PCE research has been instructive (Cunha et al., 2024). Both have been implicated in predicting outcomes, with convincing evidence of a dose–response effect (Bellis et al., 2014; Narayan et al., 2018). Given the corpus of evidence that ACEs present as risk factors for a range of criminogenic and wellbeing outcomes (Braga et al., 2018) and that PCEs may predict more favourable outcomes (Almeida et al., 2023), understanding the link between the two, not as parallel activities, but as co-occurring experiences, is a public health research priority. Given the limitations found with additive models of resilience and the difficulties associated with varying ACE and PCE thresholds, there is a case for understanding the interaction between the two to enhance our understanding of population-level needs and more accurately meet those needs (Kowalski et al., 2022).

While the research on PCEs is novel, and remains in its infancy (Han et al., 2023), the area is burgeoning. This is in part due to the development of scales such as the Benevolent Childhood Experiences (BCE) scale (Narayan et al., 2018) that has enabled a standardised approach to data collection, and has been observed most commonly in the area of mental health (Cunha et al., 2024; Han et al., 2023). Few studies have examined multiple outcomes, including criminogenic factors, such as educational exclusion, substance use and mental health. Further, those studies that have examined the relationship between ACEs and PCEs, and their predictive potential, have tended to focus on one of these, often adjusting for one when analysing the other (Cunha et al., 2024). The PCE:ACE ratio approach addresses several gaps. First, it responds to the challenge set by Crandall et al. (2020) to test new and novel ways of testing the association between PCEs, ACEs and outcomes. Several notable academics have acknowledged the idea of balance between PCEs and ACEs (Baglivio & Wolff, 2021; Bethell et al., 2019). Indeed, Cunha et al., (2024:2) note that the effect of PCEs on positive outcomes *‘…may be more pronounced when the number of PCEs outweighs the number of ACEs’,* however, few have operationalised its measurement. This study goes some way to addressing that gap and it is for future research to enhance further. Second, it recognises that PCEs and ACEs do not tend to be observed in a linear way, but rather, individuals have unique combinations of both that relate to outcomes (Han et al., 2023). Third, the ratio approach seeks to address this by combining the two into a single metric that enables us to quantify that balance (or imbalance between the two to provide a more coherent, concise and integrated indicator of both positive and negative experiences during childhood. Thus, this approach improves the predictive power of studies seeking to answer questions regarding the complex interaction between the two types of exposure and outcomes. Importantly, then, this PCE:ACE ratio should not be interpreted as a measure of resilience per-se. Rather, it provides a parsimonious indicator of the balance between protection and adversity at a population level, complementing, rather than substituting, process-oriented and developmental models of resilience.

This lens, characterised as one of seeking to understand the balance, is an approach through which we can understand exposure and outcomes. It is an approach that aligns with the evidence that regardless of the number of ACEs experienced, PCEs protect against a range of negative outcomes and promote healthy development and safe environments (Crandall et al., 2019). While previous studies have explored similar approaches (e.g., Kowalski et al., 2022), composite measures assume equal weighting of both PCEs and ACEs, something that is not sufficiently empirically verified. The compositive approach also makes interpretations more difficult, as the two constructs bleed into one another. For instance, a score of ‘0’ could mean many ACEs and many PCEs, or it could mean neither. This study is one of a few to consider the utility of a ratio of PCE:ACE and the association between that ratio and criminogenic and wellbeing outcomes. By adopting this, the ratio approach retains the single identities of both PCEs and ACEs, while calculating their relative balance for more accessible interpretation. It also allows for flexible grouping with communicative potential. The Challenge Model of Resilience is useful conceptual framework and offers another perspective for understanding the interplay between PCEs and ACEs. The ratio approach, however, is distinct insofar as it does not require even a moderate amount of adversity to produce positive outcomes; focuses on protective capacity rather than stress-inoculation; and, unlike the Challenge Model, the ratio approach offers a clearer approach to quantification, measurement, and testing.

While one objective was to explore the feasibility of transforming the two scores into a single ration that could be analysed as a raw ratio, categorised into groups, and log-transformed for statistical modelling, another was to assess the extent to which balance (or imbalance) predicted positive (or negative) outcomes. Despite quantifying the relationship differently, we observed similar patterns in previous research. In each of the outcomes measured, the direction of influence was as predicted. Less balance that moved in favour of positive childhood experiences predicted more positive outcomes. Only one outcome was no longer significant in the modelling (no formal qualifications). While previous research suggests that greater levels of PCEs are associated with better cognitive functioning (Crandall et al., 2019), and engagement in higher education (Feiler et al., 2023), our finding is not entirely clear, however, most effect sizes were small. It may be that it is the nature of the analysis and the ratio that accounts for the difference, but it also speaks to earlier research that implied that while the balance may favour some outcomes, it could be the case that other outcomes are not entirely explained by PCEs, ACEs or the combination of the two (Johnson et al., 2022). Each of the other outcomes, however, was significant at the *p* <.001 levels, with some areas, such as incarceration (Baglivio & Wolff, 2021) almost 99% less likely to be observed among the high PCE:ACE ratio group.

While the ratio approach has promising utility, it does not fully capture the complexity or heterogeneity of ACE and PCE profiles. Its simplicity is both a strength and a constraint; further analytic strategies, including moderation and threshold testing, are needed to isolate whether PCEs buffer adversity or have independent promotive effects.

This study implies several implications for both policy and practice. From a policy perspective, the insights add to the importance of developmentally appropriate opportunities, positive social connections and supports (Cullen, 1994), and pro-social activities that could be available at a universal level. Indeed, if the outcomes are of public health concern, then preventative efforts such as these could be transformative at a population level. Despite the burden on many public systems, investing in good youth work provision, creating the conditions in schools that are not only conducive to learning, but facilitate trusting and caring relationships, ensuring that adequate spaces are available for children to feel safe and to have fun, could have a longer term ripple effect that could benefit mental health and criminal justice systems. As noted by Han et al., (2023:10), while *‘the presence of adversity does not prevent PCEs from occurring…they are often somewhat related. Higher levels of adversity may be related to the lower likelihood of PCEs, particularly if the source of adversity and PCEs stem from the same people or sources.’* While the ACE literature has tended to focus on familial adversities (Madigan et al., 2023), this study implies the need to understand the wider context of children’s lives, including extra-familial adversity (Pace et al., 2022) and importantly, the protective potential of systems outside of the family context who can provide a buffering, or even remedial effect, in the context of harm.

While there is significant merit in identifying those in acute need through enhance screening (see Duffy et al., 2021), this study implies that there could be utility in transforming risk-based screening into needs-based screening. If we suspect that the balance in favour of PCEs, particularly in the absence of safety, screening tools could be refined to account for the presence (or absence) of the things that are required for healthy development.

## Conclusions

Building on the decades of research around resiliency, social support and recovery (Wolke et al., 2025), this study, while distinct from each, complements and adds to the literature by providing one response to what Seidman and Pederson (2003) referred to as the need for *‘relativism’*-understanding the co-occurring balance between positive and negative experiences. As such, this study demonstrates both a departure from and addition to these previous studies. Building on previous research, this study set out to find a way to capture the contexts that characterise more positive outcomes. Departing from pure additive models, or approaches that seek to capture individuals in their totality, andat all times of their lives (Masten & Powell, 2003), this approach set out to explore something different.

This study showed that it is feasible to combine both a measure of childhood adversity and a measure of positive childhood experiences that retains both constructs but that also enables statistical analysis of both simultaneously. Rather than simply an accumulation of both operating in parallel, the ratio measures balance and requires neither adversity nor PCEs to be present as a pre-requisite. In measuring experiences in this way and at one point in time the added value of the ratio metric is that it allows us to capture the relational balance between the two and to use this balance as the measure of how well or not the population, rather than individuals, are doing. Indeed we found credible evidence that a balance tipped in favour of a higher PCE:ACE ratio is associated with more positive outcomes and modelling predicts significantly lower criminogenic factors and better mental health.

While this study was undertaken in the Northern Ireland, with a sample who have lived in a context with distinctive socio-historical characteristics (e.g., conflict) that could shape ACE and PCE experiences, the analytic aim of this study was to explore population balance, not regional comparison of the types of experiences that define adverse and positive childhoods. Methodologically, the PCE:ACE ratio approach has the potential to transform how ACE research is conducted and communicated. Rather than regressing risk or protective/promotive factors on outcomes, as might be done with resiliency research (Wolke et al., 2025), the ratio provides a single metric on which to model outcomes. It shifts the focus from a deficit-based inquiry to one that retains the ability to include adversity in the analysis, while focusing on strengths. Rather than the individually oriented and often diagnostically concerned resiliency tools, this approach provides an adaptable framework for capturing and understanding a dimension of social health, but at a population level. As Ungar (2013: 260) notes, *‘[c]ohort studies show that the capacity of a population to overcome adversity is often related to the capacity and willingness of their societies to provide resources’,* the ratio offers a heuristic to operationalise how measuring such balance is achieved.

The ratio-based approach is an integrated metric that captures the balance of strengths and adversity directly, is empirically useful as it provides strong predictors that elevates the focus on ACEs or PCEs independently, is resilience focused, is flexible where it can be tested across risk levels and is policy relevant with intuitive thresholds. The metric responds to the gap in empirical studies around healthy population development that have tended to focus on the role of variables at the expense of understanding the simultaneous interaction between two the sides of the equation. Future research should expand this analysis with different populations and different outcomes. There is potential to examine the thresholds used (high, moderate, low) to determine if the cut-offs proposed here at the most optimal. While the study used a single ratio, future research could expand on this to explore whether or not PCE-ACE sub-groups can be used to determine the optimum balance and type of positive and adverse childhood experience, and whether they enhance the predictive power of this approach. Finally, and importantly, this study does not propose that those who transition out of childhood with a lower PCE:ACE ratio are deterministically at risk of offending or poorer mental health as it neither claims to capture the entirely of an individual’s circumstances nor all of their lifespan. Indeed, it does not even propose to be used as a diagnostic or individual screening tool. Instead, the ratio puts positive and negative experiences on the same scale and responds to a simpler question: are the childhood experiences of the population tipped in favour of protection over adversity? If the latter, the tool can be useful not only for capturing the health of a population but also as a metric of population health improvement.

While this shows an association, the poorly understood causal mechanisms require further consideration. Further, while this study points towards efforts to enhance universal or primary prevention efforts (e.g., via increasing support for positive youth development activities and youth services), it does not negate the need for specialist care for some of those who experience a burden of ACEs and an absence of PCEs. Instead, this study points to the utility of framing the pipeline between early experiences and positive health/criminal justice outcomes within a wider public health context, with PCEs nested within primary prevention alongside other efforts that will be required at secondary and tertiary levels.

### Limitations

While the PCE:ACE ratio offers an intuitive and integrative approach to examining the balance between adversity and support, it may oversimplify the relationship between these constructs. Individuals with the same ratio can have vastly different profiles of ACE and PCE exposure, for instance, 6 ACEs and 6 PCEs versus 2 ACEs and 2 PCEs yield the same ratio but likely reflect different lived experiences and risks. The ratio also constrains interpretation of the unique or interactive effects of ACEs and PCEs. As such, while useful as a heuristic, it should be treated as exploratory. The insights in this exploratory study should be taken with some degree of caution. Given that some of the key outcomes explored here effect a small proportion of the population, the numbers included in the between groups analyses become even smaller when disaggregating by PCE:ACE groups. Future research could increase sub-group sample size and should complement this approach with moderation or threshold analyses to clarify these dynamics more precisely. Future research should also explore ways that this lens could provide concrete direction to policy-makers, nesting negative mental health and offending outcomes within a wider public health framework, and provide preventative hope for those whose experiences are laden with adversity over positive childhood experiences. However, it must finally be noted that the ratio is intended as a population-level heuristic indicator of developmental balance, not as an individual-level measure of risk.

## Data Availability

No new datasets were generated during the current study.
